# Effects of Prenatal Social Stress and Maternal Dietary Fatty Acid Ratio on Infant Temperament: Does Race Matter?

**DOI:** 10.4172/2161-1165.1000167

**Published:** 2014

**Authors:** Kelly J. Brunst, Michelle Bosquet Enlow, Srimathi Kannan, Kecia N. Carroll, Brent A. Coull, Rosalind J. Wright

**Affiliations:** 1 Kravis Children’s Hospital, Department of Pediatrics, Icahn School of Medicine at Mount Sinai, One Gustave L. Levy Place, New York, NY 10029, USA; 2 Boston Children's Hospital, Department of Psychiatry, Program for Behavioral Science and Harvard Medical School, Department of Psychiatry, 300 Longwood Avenue, AT-120.3, Mailstop BCH 3199, Boston, MA 02115, USA; 3 Human Nutrition and Dietetics, College of Agricultural Sciences, Southern Illinois University, 1205 Lincoln Drive, Carbondale, IL 62901, USA; 4 Department of Pediatrics, Vanderbilt University School of Medicine, 1161 21st Avenue, Nashville, TN 37232, USA; 5 Department of Biostatistics, Harvard School of Public Health, 655 Huntington Avenue, Boston, MA 02115, USA; 6 Mindich Child Health and Development Institute, Icahn School of Medicine at Mount Sinai, 1428 Madison Avenue, New York, NY 10029, USA

**Keywords:** Psychosocial stress, Temperament, Fatty acids, Ethnicity, Race

## Abstract

**Background:**

Infant temperament predicts a range of developmental and behavioral outcomes throughout childhood. Both maternal fatty acid intake and psychosocial stress exposures during pregnancy may influence infant temperament. Furthermore, maternal race may modify prenatal diet and stress effects. The goals of this study are to examine the joint effects of prenatal diet and stress and the modifying effects of race on infant behavior.

**Methods:**

Analyses included N=255 mother-infant dyads, primarily minorities (21% Blacks; 42% Hispanics), enrolled in an urban pregnancy cohort. Maternal prenatal stress was indexed by a negative life events (NLEs) score on the Crisis in Family Systems-Revised survey. Prenatal total daily intakes of polyunsaturated fatty acids (PUFAs) (n3, n6) were estimated from a food frequency questionnaire; n3:n6 ratios were calculated. Mothers completed the Infant Behavior Questionnaire-Revised (IBQ-R), a measure of infant temperament, when the children were 6 months old. Three commonly used dimensions were derived: Orienting & Regulation, Extraversion, and Negative Affectivity. Associations among prenatal stress, maternal n3:n6 ratio, and race/ethnicity on infant temperament, controlling for maternal education and age and child sex, were examined.

**Results:**

Among Blacks, prenatal stress effects on infant Orienting & Regulation scores were modified by maternal n3:n6 ratios (p=0.03): As NLEs increased, lower n3:n6 ratios predicted lower infant Orienting & Regulation scores, whereas higher n3:n6 ratios attenuated the effect of prenatal stress. There were no main or interaction effects predicting Extraversion or Negative Affectivity.

**Conclusions:**

An optimal PUFA ratio may protect the fetus from stress effects on infant behavior, particularly among Blacks. These findings may have implications for later neurodevelopment and social functioning predicted by early temperamental characteristics.

## Introduction

Infant temperament, defined as constitutionally based individual differences in reactivity and self-regulation [1], predicts emergence of later childhood behavioral and emotional problems, including poor executive functioning abilities, diminished social responsiveness, and internalizing/externalizing symptoms [2–4]. Data suggest that prenatal factors may influence infant temperamental profiles, potentially having long-term impact on child developmental outcomes [5–7]. Exposure to prenatal stress has been identified as a robust programmer of child behavioral outcomes [5,8]. Prenatal diet has also been implicated in child neurodevelopment [9]. To date, little work has explored the joint effects of prenatal stress and diet on child temperament, though evidence suggests that they may have independent and synergistic effects, operating through common mechanisms [7]. Furthermore, emerging data indicate that maternal race may modify stress and diet exposure effects, though such modification effects have not been studied in relation to infant behavior.

The brain is highly sensitive to stress in the prenatal period, with substantial changes in structural growth and connectivity occurring in fetal life [10]. Gestation represents a time of fetal neuron proliferation, differentiation, migration, and aggregation, processes that are genetically determined, epigenetically directed, and environmentally influenced [10], with maternal stress being a potent environmental influence. Poor behavioral outcomes as a result of maternal prenatal stress are supported by animal studies linking maternal stress with adverse fetal brain development, such as alterations in the hippocampus that result in increased vulnerability to later neuropathologies [11,12]. Epidemiological studies provide further evidence that maternal stress during pregnancy is associated with increased incidences of difficult temperament in infancy and childhood behavioral problems [6,13].

Prenatal psychosocial stress and prenatal diet may influence fetal brain development via common central mechanisms. Prenatal stress promotes oxidative stress and thus oxidative damage [14–16] and inflammation both systemically and locally in the developing brain [17,18]. During pregnancy, an imbalance in detoxification capacity at the level of the placenta may translate into enhanced oxidative stress in fetal tissues, including the central nervous system [19]. Prenatal diet may also influence oxidative stress and inflammation. Essential fatty acids obtained largely through dietary sources, specifically polyunsaturated fatty acids (PUFAs), have known antioxidant and anti-inflammatory properties that impact placental functioning [20] and play a central role in the development and functioning of the brain [21,22]. Epidemiological studies suggest that it is the ratio between maternal dietary n-3 and n-6 PUFA intakes that is critical for a child’s brain development during fetal life, with a higher proportion of n-3 PUFAs compared to n-6 PUFAs being beneficial [9,23,24].

Overlapping lines of evidence suggest that race/ethnicity may influence associations among prenatal stress, prenatal diet, and infant temperament. First, research suggests that racial/ethnic minorities may be differentially vulnerable to stress-elicited health effects due to increased exposure to adverse events across their life course, reduced resources that buffer stress effects (e.g., coping abilities, perceived social support), and ongoing discrimination stress [25]. Second, for reasons that remain largely unknown, oxidative stress and inflammation vary by race and differences in stress-elicited physiological responses to a number of environmental stimuli vary by race. For example, Blacks are not only more likely to exhibit elevated oxidative stress and inflammation [26], but are also more likely to experience these physiological changes in response to environmental stimuli such as tobacco smoke and air pollution [27–29]. Exposure to passive tobacco smoke has been linked to oxidative DNA damage in Black but not White adults [27]. Similar findings have been reported for exposure to air pollutants, including ozone and ambient particulate matter [28,29]. Moreover, compared to Whites with low antioxidant intakes, Blacks with low antioxidant intakes exhibit higher leukocyte counts and higher levels of high sensitivity C-reactive protein [30], suggesting a greater degree of inflammation. The few studies that have looked at racial/ethnic differences in response to social stress have shown Blacks to exhibit faster biological aging and blunted cortisol responses [31–33], outcomes which in turn are associated with enhanced oxidative stress and inflammation [34–36].

We leverage an ethnically diverse sample to address a number of these research gaps. The goals of the current study were to (a) test the joint effects of prenatal psychosocial stress exposures and prenatal PUFA n3:n6 ratio on infant temperament and (b) to test the modifying effects of maternal race/ethnicity on prenatal stress and diet associations with infant temperament. We hypothesized that an increased PUFA n3:n6 ratio attenuates the effects of increased maternal stress on infant behavior and that this effect is particularly strong among racial/ethnic minorities.

## Methods

Study sample: Mother-infant pairs were from the PRogramming of Intergenerational Stress Mechanisms (PRISM) study, a prospective pregnancy cohort originally designed to examine how perinatal stress influences respiratory health in children. Between March 2011 and March 2013, 281women were recruited from prenatal clinics during the first or second trimester (<28 weeks gestation) from the Beth Israel Deaconess Medical Center (BIDMC) and the East Boston Neighborhood Health Center. Recruitment sites were chosen given desired heterogeneity in sociodemographic and racial/ethnic characteristics. Eligibility criteria included: (a) English- or Spanish-speaking; (b) age >18 years at enrollment; and (c) singleton pregnancies. Mothers who endorsed drinking ≥ 7 alcoholic drinks/ week prior to pregnancy recognition or any alcohol following pregnancy recognition were excluded due to increased risk for neurodevelopmental problems [37]. For the current analyses, women identifying as multi-racial (n=26) were excluded to allow comparison among Whites, Hispanics and Blacks, leaving the final sample size of 255. Procedures were approved by the relevant institutions’ human studies committees; written consent was obtained in the subject’s primary language.

## Procedures and measures

### Maternal prenatal negative life events

The Crisis in Family Systems-Revised (CRISYS) survey was administered in pregnancy within 2 weeks of enrollment to assess exposure to negative life events over the past six months. The CRISYS is suitable for lower-income populations, has good test/retest reliability, and has been validated in English and Spanish [38]. The survey encompasses 11 domains (financial, legal, career, stability in relationships, medical issues pertaining to self, medical issues pertaining to others, safety in the community, safety in the home, housing problems, difficulty with authority, discrimination), with multiple items in each domain. Women rated endorsed items as positive, negative, or neutral experiences. Because research suggests increased vulnerability when experiencing events across multiple domains [39], the number of domains with one or more negative events endorsed was summed to create a negative life events (NLEs) domain score (possible range = 0–11; range in current sample = 0–9), with higher scores indicating greater stress.

### Dietary intakes

Information on maternal dietary and supplemental intakes was obtained at enrollment using the Block98 food frequency questionnaire (FFQ) [40,41], modified to include a more extensive list of fish and seafood items [42]. The measure has been validated in multicultural populations [41,43]. Details of the dietary assessment and processing of the FFQ data in this cohort have been previously described [44]. Briefly, using a food composition database, maternal energy-adjusted nutrient intakes of total n6 PUFAs [as the sum of linoleic acid (LA,g/d) and arachidonic acid (ARA,g/d)] and total n3 PUFAs [as the sum of alpha-linolenic acid (ALA,g/d), eicosappentaenoic acid (EPA,g/d), docosapentaenoic acid (DPA,g/d), and docosahexaenoic acid (DHA,g/d)] were estimated. Notably, Western diets are characterized by deficient intakes of n3 fatty acids and excessive amounts of n6 fatty acids, a combination that has been shown to promote the pathogenesis of many diseases [45]. Therefore, the n3:n6 fatty acid ratio was calculated and used in the analyses with a higher ratio indexing more anti-inflammatory/antioxidant properties.

### Infant temperament

Infant temperament was ascertained at 6 months of age using the Infant Behavior Questionnaire-Revised (IBQ-R). The 191-item IBQ-R [46] has been demonstrated to have good reliability and validity in 6 month olds [47] and has been used in English- and Spanish-speaking populations [48,49]. The questionnaire was administered as an interview, and the mother was shown a card with response choices. Mothers rated the frequency that their children engaged in specific day-to-day behaviors in the prior week using a 7-point scale, with responses ranging from 1 (‘never’) to 7 (‘always’). Scores were summed across items to create multiple subscales assessing different temperamental dimensions. Subscales were then summed to create three commonly used domain scores, with higher scores indicating greater levels of that domain: Extraversion, Orienting & Regulation, and Negative Affectivity [2,4]. The Extraversion domain scale consists of the subscale dimensions of Activity Level, Approach, High Intensity Pleasure, Perceptual Sensitivity, Smiling/Laughter, and Vocal Reactivity. The Orienting & Regulation domain scale consists of the dimensions of Soothability, Duration of Orienting, Low Intensity Pleasure, and Cuddliness. The Negative Affectivity domain scale consists of the subscale dimensions of Fear, Distress to Limitations, Sadness, and Falling Reactivity/Rate of Recovery from Distress. Low to moderate correlations were observed among the domain scores: r=0.22 (p<0.001) for Extraversion and Negative Affectivity, r=0.42 (p<0.001) for Extraversion and Orienting & Regulation, and r=0.02 (p=0.66) for the Negative Affectivity and Orienting & Regulation.

## Data analysis plan

Data analyses proceeded in several steps. First, differences in sample characteristics, negative life events, dietary intakes, and infant temperament by race/ethnicity were examined (Table 1). Where appropriate, a Chi-square test or Fisher’s exact test was performed to investigate differences in the distribution of child sex and maternal education level by race, respectively. One-way analysis of variance (ANOVA) and Kruskal Wallis non-parametric ANOVA for non-normal distributions were used to test for differences in maternal age, maternal stress (NLEs), PUFA intakes, n3:n6 ratio, and IBQ-R scores by race/ethnicity. Significant tests were followed by a Mann-Whitney-Wilcoxon test with Bonferroni adjustment for multiple comparisons to compare scores between specific racial/ethnic groups. Linear regression was used to assess the association between maternal NLEs and IBQ-R scores; each IBQ-R domain score was modeled separately. Model covariates were identified based on previously published literature suggesting relationships with maternal stress and child behavioral outcomes: sex, race/ethnicity (White, Black, Hispanic), maternal education (did not complete high school/high school or greater), and maternal age (years) [50,51]. First-order interaction products between NLEs x n3:n6 and NLEs x race/ethnicity were examined for each IBQ-R outcome. Since the ratio of PUFAs is important for brain development [9,24] and since race/ethnicity may be an independent risk factor for enhanced stress-vulnerability, effect modification of stress by n3:n6 ratio on IBQ-R scores were examined by race-stratification. If significant racial differences were observed, interaction terms for the three-way interaction (NLEs x n3:n6 x race) were added to the regression model. P-values<0.1 for interaction terms were considered significant as per prior research [52,53]. All other tests assumed a two-sided alternative hypothesis and a 0.05 significance level and were conducted using SAS 9.3 (SAS Institute Inc., Cary, NC, USA).

## Results

### Descriptive data

Characteristics of the sample are detailed in Table 1. Differences in maternal education were observed across racial categories, with 96% of those having less than a high school degree being minorities (n=82), the majority being Hispanic (n=67). The Black and Hispanic women were significantly younger than the White women (p=0.002). Distributions of NLEs, PUFAs intakes, n3:n6 ratios, and IBQ-R outcomes in the total cohort and by race/ethnicity are also outlined in Table 1. Blacks reported experiencing a greater number of NLEs compared to Whites (p<0.0001) and Hispanics (p=0.002). Whites and Blacks had significantly lower intakes of total n3 and n6 PUFAs as well as lower n3:n6 ratios compared to Hispanics (all p-values<0.001). Infants of Hispanic women scored higher in Orienting & Regulation compared to infants of White women (p=0.0001); infants of both Hispanic and Black women scored higher in Extraversion than infants of White women (p=0.004). Infants of Hispanic women scored higher in Negative Affectivity compared to infants of Black (p<0.0001) and White women (p=0.0003).

### Main effects of NLEs

Unadjusted associations between maternal NLEs and the three IBQ-R domain scores did not reach statistical significance (all p-values>0.20). Similarly, after adjustment for covariates, maternal NLEs did not predict Extraversion (β=0.01; 95% CI −0.03, 0.05; p=0.60) or Orienting & Regulation (β= −0.03; 95% CI −0.06, 0.01; p=0.17). A marginally significant positive association was observed between maternal NLEs and Negative Affectivity (β=0.03; 95% CI −0.006, 0.06; p=0.09).

### Two- and three-way interactions

There was no evidence of effect modification by n3:n6 ratio or race/ ethnicity on NLEs (i.e., individual two-way interactions were not significant, all p-values>0.25). Given the study’s objective to examine racial differences in associations among prenatal stress, diet, and infant temperament, race-stratified analyses were conducted to examine the synergistic effect of maternal NLEs and n3:n6 ratio on the three IBQ-R domains. These results can be found in Table 2. Among Blacks, the interaction between maternal NLEs and n3:n6 ratio significantly predicted infant Orienting & Regulation (β= −3.63; 95% CI −7.07, −0.20; p=0.03). The interpretation of this interaction is that as NLEs increased, the infants of mothers with low n3:n6 ratios exhibited lower Orienting & Regulation scores; higher maternal n3:n6 ratios attenuated the effect of increasing NLEs on infant Orienting & Regulation scores. This effect was not observed among infants of White or Hispanic women. The likelihood ratio type 3 analysis for the three-way interaction (i.e., NLEs x n3:n6 x race) was significant in the model predicting infant Orienting & Regulation (p=0.08). All other three-way interactions were not significant (all p-values>0.25).

## Discussion

This study is the first prospective analysis suggesting that the effects of prenatal maternal stress on infant temperament may be modified in a race-dependent manner by nutrients shown to reduce fetal vulnerability to oxidative stress and inflammation. As hypothesized *a priori*, the findings suggest that the effects of prenatal social stress exposure on infant temperament, specifically the domain of Orienting & Regulation, were modified by maternal prenatal n3:n6 ratio in Black but not White or Hispanic infants. Specifically, Black infants whose mothers were exposed to increased social stress and had lower n3:n6 ratio intakes in pregnancy had lower scores on Orienting & Regulation; higher n3:n6 ratio intakes appeared to ameliorate prenatal stress exposure effects.

Interestingly, the effects of maternal prenatal social stress and diet were specific to infant Orienting & Regulation, which included behaviors such as the infant’s attention to and/or interaction with a single object for extended periods of time [46]. A recent study found similar evidence of prenatal maternal stress effects on infant persistence and attention [5]. Maternal stress, together with a lower maternal n3:n6 PUFA ratio during pregnancy, which are both posited to be detrimental to many aspects of brain development [9], may have particular impact on brain areas involved in the ability to orient and self-regulate, such as the prefrontal cortex [54]. Future research needs to explicate the brain areas most susceptible to the joint effects of prenatal stress and diet.

Potentially modifiable environmental effects on infant orienting and regulation behaviors are of particular interest for two reasons. First, during infancy, the natural propensity to orient provides a means of soothing. Derauf et al. [55] observed that infants who exhibit increased soothability and greater durations of orienting, both scales in the Orienting & Regulation domain of the IBQ-R, were less likely to display externalizing and internalizing behavior problems at three years of age. Second, research suggests that Orienting & Regulation may contribute to later effortful control [56]. Low effortful control has been linked to a variety of mental health problems in later childhood and adolescence [4,57–59], thus having implications for long-term psychopathology risk.

The current findings indicate that prenatal dietary interventions may ameliorate some of the negative impact of prenatal stress exposures on infant behavior, particularly among high-risk groups, such as lower-income Black women and infants. Low-income Black women are disproportionately exposed to chronic and extreme social stressors [60,61] and may experience more intense physiological consequences (e.g., oxidative stress, inflammation) for reasons not currently fully understood [27–30]. Mechanistic studies, perhaps exploring genetic and epigenetic susceptibility, are needed to fully understand the racial differences observed. The implications of the n3:n6 ratio (i.e., shift towards an increase in n6 and a decrease in n3 PUFAs) and the mechanisms involved with respect to effects on temperament among Black infants are also not entirely clear. While studies suggest greater maternal intake of n3 fatty acids and less intake of n6 fatty acids is beneficial to a child’s neurodevelopment [9], this is the first study to look at the effects of maternal PUFA intakes and stress on early-life neurobehavioral outcomes in a racially diverse population. We acknowledge that continued research is needed to better understand the complex three-way relationship between prenatal maternal stress, PUFA intakes and race; yet, the adverse effects of increased stress in conjunction with low n3:n6 ratios observed in our study suggest the potential for dietary intervention in at-risk populations (i.e., Blacks).

This study has a number of strengths, including the prospective design and the largely racial/ethnic minority, lower-income sample. Limitations include reliance on maternal report for ratings of infant temperament, which may be subject to reporting bias as mothers with higher stress may report more adverse behaviors in their children [62]. However, the IBQ-R provides a measure of the infant’s general response/behavior patterns, as opposed to a snapshot of behavior that would be observed in a laboratory assessment, which is susceptible to situational factors. Furthermore, a strong relationship between observational measures of infant behavior and parental report via the IBQ-R has been established [47]. Also, maternal bias would unlikely influence reporting of nutrition, stress, and infant behavior in a consistent way that would influence associations among the variables and thus account for the reported findings. Longitudinal follow-up of these children will allow for more definitive behavioral phenotyping. Food and supplemental intakes were based on subject recall and therefore vulnerable to under- or over-estimation. Notably, this study used the modified Block98 FFQ, which has been validated in multicultural and periconceptional populations [40,41]. Moreover, FFQs are widely used to obtain estimates of usual dietary intakes over long periods of time (e.g., months), including during pregnancy [63], and reported intakes of PUFAs via FFQs in particular correlate with biological markers [64].

## Conclusion

The findings suggest that prenatal social stress effects on infant temperament may be modified by maternal prenatal diet, particularly in Black infants. Adequate intakes of nutrients with antioxidant and anti-inflammatory effects may be important for protecting the fetus from environmentally-induced oxidative stress/inflammation, for which Blacks are arguably most at risk. Thus, these findings raise the question of whether maternal nutritional interventions, particularly around fatty acids, could reduce the deleterious impact of maternal stress on infant neurodevelopment by enhancing favorable behavioral phenotypes during infancy. While efforts to reduce exposure to chronic stress are arguably the ideal intervention in this context, dietary interventions may be more feasible to implement in the short-term. That is, effective reduction in cumulative stressors to which lower income, racial/ethnic minority populations are exposed requires more long-term strategies up to and including at the policy level.

## Figures and Tables

**Table 1:** Sample characteristics and distribution of negative life events, PUFA intakes, n3:n6 ratio, and IBQ-R scores in the total sample and stratified by race/ethnicity

	Total (n = 255)		Whites(n = 95)		Blacks (n = 53)		Hispanics (n = 107)		
	n	%	n	%	n	%	n	%	p§
Maternal education < HS^a^	85	33	3	3	15	29	67	63	<0.001^f^
Child sex (male)	128	50	50	53	28	53	50	47	0.61
	Mean (Median)	SD^b^	Mean (Median)	SD	Mean (Median)	SD	Mean (Median)	SD	p§
Maternal age (years)	30 (31)	5	32 (32)	4	29 (30)	6	29 (30)	6	0.002
NLEs^c^	2.02 (1.00)	1.89	1.65 (1.00)	1.60	2.97 (3.00)	1.96	2.13 (1.00)	2.00	<0.001
PUFAs^d^									
Total n3 (g/d)	2.09 (1.86)	0.84	1.81 (1.72)	0.48	1.92 (1.77)	0.76	2.42 (2.28)	1.01	<0.001
Total n6 (g/d)	14.93 (14.36)	3.97	14.07 (14.04)	2.44	14.27 (13.19)	4.45	16.02 (15.51)	4.53	0.002
n3:n6 ratio	0.14 (0.13)	0.03	0.13 (0.12)	0.02	0.13 (0.13)	0.02	0.15 (0.15)	0.03	<0.001
IBQ-R domains^e^									
Extraversion	5.09 (5.10)	0.62	4.86 (4.85)	0.55	5.21 (5.24)	0.64	5.27 (5.29)	0.64	<0.001^g^
Orienting &Regulation	5.31 (5.30)	0.55	5.17 (5.17)	0.52	5.34 (5.23)	0.55	5.46 (5.44)	0.49	<0.001^g^
Negative Affectivity	3.64 (3.59)	0.52	3.53 (3.50)	0.39	3.43 (3.42)	0.46	3.82 (3.72)	0.58	0.0002

a High School (HS);

b Standard Deviation (SD);

c Negative Life Events (NLEs);

d total n3 polyunsaturated fatty acids and total n6 polyunsaturated fatty acids (PUFAs) ratio (n3:n6);

e Infant Behavior Questionnaire-Revised (IBQ-R);

§ p value for differences on covariates across racial/ethnic groups;

f Fisher’s exact test used due to small cell sizes;

g One-way analysis of variance (ANOVA) used to test differences in means; all other continuous variables tested using the Kruskal Wallis ANOVA (non-parametric).

**Table 2:** Race-stratified adjusted associations between maternal negative life events (NLEs) and n3:n6 ratio on infant Extraversion, Orienting & Regulation, and Negative Affectivity

Outcome	β^a^	95% CI^b^	p
**White**			
Extraversion	−0.32	−3.31, 2.46	0.82
Orienting & Regulation	0.21	−2.49, 2.90	0.88
Negative Affectivity	−0.76	−2.67, 1.16	0.44
**Black**			
Extraversion	−2.57	−6.24, 1.12	0.17
Orienting & Regulation	−3.63	−7.06, −0.20	0.03
Negative Affectivity	1.41	−1.60, 4.43	0.35
**Hispanic**			
Extraversion	0.32	−1.61, 2.25	0.74
Orienting & Regulation	−0.34	−1.83, 1.17	0.66
Negative Affectivity	−0.38	−2.19, 1.42	0.67

a Beta coefficient (β) for the statistical interaction term between NLEs and n3:n6 ratio;

b confidence interval (CI). Each model is adjusted for maternal education, maternal age, and child’s sex.
